# No longer locally extinct? Tracing the origins of a lion (*Panthera leo*) living in Gabon

**DOI:** 10.1007/s10592-017-1039-2

**Published:** 2018-02-01

**Authors:** Ross Barnett, Mikkel-Holder S. Sinding, Filipe G. Vieira, Marie Lisandra Zepeda Mendoza, Matthieu Bonnet, Alessandro Araldi, Ivonne Kienast, Alice Zambarda, Nobuyuki Yamaguchi, Philipp Henschel, M. Thomas P. Gilbert

**Affiliations:** 1 Natural History Museum of Denmark, University of Copenhagen, Øster Voldgade 5-7, 1350 Copenhagen, Denmark; 2 Natural History Museum, University of Oslo, Blindern, P.O. Box 1172, 0318 Oslo, Norway; 3 The Aspinall Foundation, Port Lympne Wild Animal Park, Hythe, Kent CT21 4PD UK; 4 Congo Program, Wildlife Conservation Society, Brazzaville, Congo; 5 Department of Biological and Environmental Sciences, Qatar University, Doha, Qatar; 6 Panthera, 8 West 40th Street, 18th Floor, New York, NY 10018 USA; 7 Institut de Recherche en Ecologie Tropicale, CENAREST, BP 842 Libreville, Gabon; 8 NTNU University Museum, 7491 Trondheim, Norway

**Keywords:** Ancient DNA, Mitochondrial genomes, *Panthera leo*, Lion, Gabon, Congo, Plateaux Batéké National Park

## Abstract

Lions (*Panthera leo*) are of particular conservation concern due to evidence of recent, widespread population declines in what has hitherto been seen as a common species, robust to anthropogenic disturbance. Here we use non-invasive methods to recover complete mitochondrial genomes from single hair samples collected in the field in order to explore the identity of the Gabonese Plateaux Batéké lion. Comparison of the mitogenomes against a comprehensive dataset of African lion sequences that includes relevant geographically proximate lion populations from both contemporary and ancient sources, enabled us to identify the Plateaux Batéké lion as a close maternal relative to now extirpated populations found in Gabon and nearby Congo during the twentieth century, and to extant populations of Southern Africa. Our study demonstrates the relevance of ancient DNA methods to field conservation work, and the ability of trace field samples to provide copious genetic information about free-ranging animals.

**Electronic supplementary material:**

The online version of this article (10.1007/s10592-017-1039-2) contains supplementary material, which is available to authorized users.

## Introduction

Lions are currently experiencing extensive population declines (Bauer and Van Der Merwe 2004; Bauer et al. 2015) throughout their African range, with the exception of those in artificially managed private reserves in the South and East of the continent. Of particular concern are those populations found in West and Central Africa, which have repeatedly been shown to be genetically distinct (Barnett et al. 2006a, b, 2014; Bertola et al. 2011, 2015, 2016; Antunes et al. 2008; Dubach et al. 2013). The total population of the West African lion (previously known as *Panthera leo senegalensis*) may be fewer than 250 mature animals, occupying a range ~ 1% of their historic extent (Henschel et al. 2014). Similarly, lions from Central Africa, within, or adjacent to, the Congo Basin, (previously known as *Panthera leo azandica/bleyenberghi/senegalensis*) may number less than 2000 individuals (Bauer et al. 2015). Given the real danger that lions face of regional extinction, any evidence of population recovery, previously unknown populations, or new migrations into former territories, has immense benefit for our understanding of lions in this area and boosting conservation morale. In this regard, Gabon, a heavily forested country in western Central Africa, has recently received attention. Until recently it was thought to have no free-ranging lions left, due to severe human persecution and historically low numbers of lions (Bauer and Van Der Merwe 2004; Nowell and Jackson 1996), and the lack of conclusive field evidence from field surveys in former lion range (Henschel 2006). Lions were therefore listed as locally extinct in Gabon, at a regional workshop held to define the current status and conservation strategies for the lion in West and Central Africa (IUCN 2006). The species is also no longer considered by national legislation in Gabon. However, prompted by the discovery of a single male lion in camera trap images from the Plateaux Batéké National Park (PBNP) in southeast Gabon (Hedwig et al. 2017), field survey work identified four possible lion hair samples. We applied ancient DNA techniques that are appropriate for dealing with the low levels of DNA present in hair shafts to this physical evidence in order to (i) recover genetic information from the samples, (ii) confirm their species identities, (iii) infer the relationship between this Batéké lion and other proximal lion populations, and (iv) suggest potential scenarios that would explain the appearance of this individual in Gabon.

## Materials and methods

### Sample collection

**Fig. 1:**
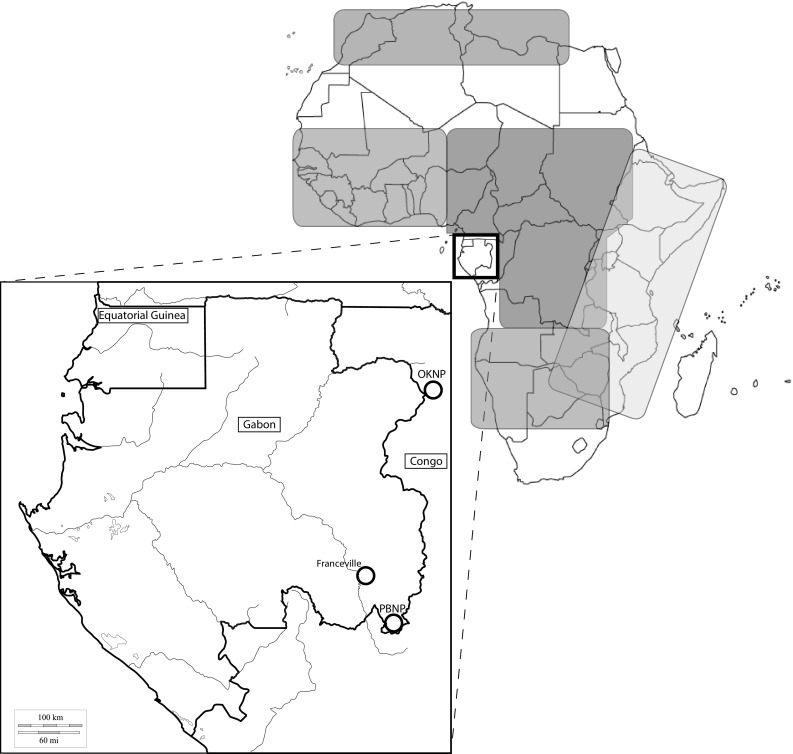
Map of Central Africa, showing Gabon and Congo, with locations mentioned in the text. *OKNP* Odzala-Kokoua National Park, *PBNP* Plateaux Batéké National Park. Also shown are approximate distributions of the main maternal haplotype lineages as shown in Barnett et al. (2014). Central lineage = Red; Western lineage = Orange; Eastern lineage = yellow; Southern lineage = pink; Northern lineage = blue. (Color figure online)

**Fig. 2:**
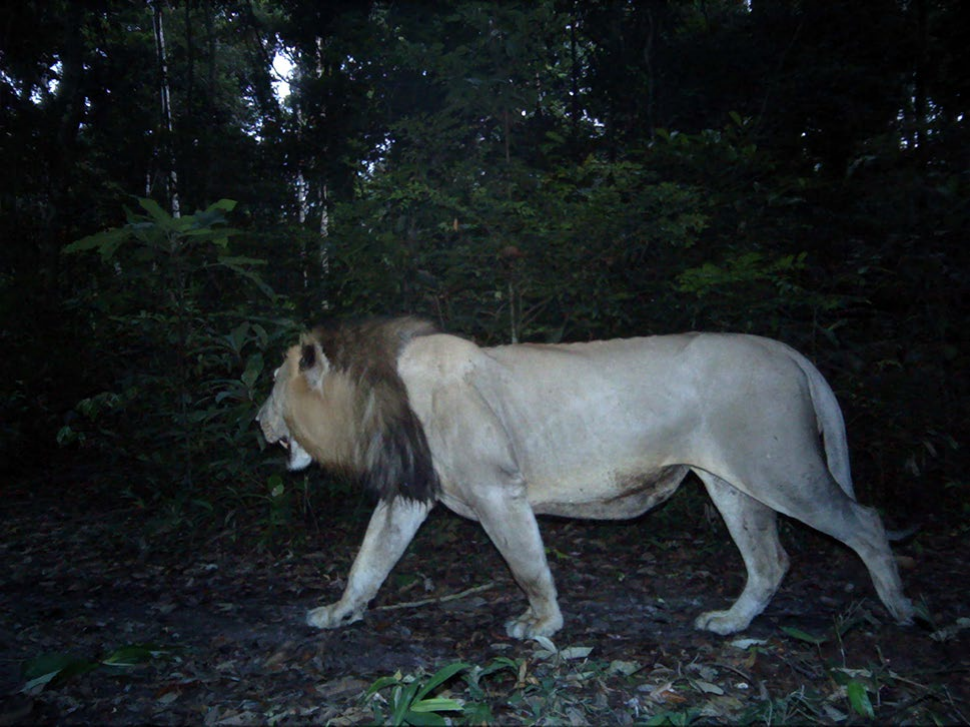
Camera trap image of the PBNP male lion taken in 2016

The Gabon male was first recorded in PBNP (Fig. 1) in January 2015, by camera traps set up as part of a study on chimpanzees (*Pan troglodytes*) conducted by the Pan African Programme of the Max Planck Institute for Evolutionary Anthropology (MPI-EVA PanAf) (Hedwig et al. 2017). The discovery sparked hope about the potential presence of a breeding population, almost 20 years after the last confirmed record of breeding lions in Gabon (Henschel 2006). However, additional camera trap surveys in a wider 400-km^2^ area encompassing the initial photo-capture site only produced repeated records of the very same male (Fig. 2). After more than 1 year of continuous camera trapping it was concluded that only this one male was present in the park. Following this conclusion, the Gabonese National Park Agency (ANPN) expressed interest in evaluating the possibility of establishing a breeding population through the importation and release of wild-caught females, using this Batéké male as one of the founders. This required the collection of samples from this male for genetic analysis, to determine his genetic makeup and to identify a suitable source population for the capture of females for inclusion into a founder population.

Sample collection was not straightforward, as the Gabon male is extremely wary and has never been directly observed. PBNP cannot be accessed by vehicle, and it was therefore also not possible to use a biopsy dart from the safety of a vehicle, even if the lion could be lured in using bait or audio playbacks (cf. Ogutu and Dublin 1998). We therefore decided to collect non-invasive samples, by searching the park for putative lion hairs and faeces. To increase our chances of finding samples from one single animal in the 2000 km^2^ national park, we intensified searches at sites where the lion was photo-trapped. Furthermore, whenever the lion was heard vocalising, which was usually at night, we travelled to the rough location of the vocalisation the following day, and stashed a wildlife caller in a tree to try to attract the animal to that specific spot. For this, we used a commercially available FOXPRO (FOXPRO INC; Lewistown, PA, USA) Snow-Crow Pro 5 game call system with a built-in 50 W amplifier and two high-performance conical speakers. The caller was programmed to broadcast the roar of a single female lioness continuously, with a 1 h 10-min pause between series of four bouts of roaring. We additionally sprayed the base of the tree in which the caller was stashed with urine from female lions, collected from captive animals in Gabon’s capital Libreville, to trigger marking behaviour in the male. Over the course of 2016, we managed to collect five individual hair samples thought to be from the lion, one of which was collected at a camera trap site where the lion was recorded, and four of which came from a single call-up station. All hairs appeared to be long mane hairs, and were found stuck in the bark of trees, which the male likely cheek-rubbed. All hair was stored in 50 ml sampling tubes with silica beads.

To compare the genetic makeup of the Batéké male to populations historically occurring in this landscape, we also tried to source older samples of lions killed in Gabon and neighbouring Republic of Congo in the twentieth century. Among the last confirmed lion records from the Republic of Congo, was the case of two males shot in a hunting concession now part of Odzala-Kokoua National Park (Dowsett 1995). The skins and skulls of these lions are still in possession of the Congolese Ministry of the Environment. With their kind permission, we collected a small (1 × 1 cm) skin sample from one individual. The skin piece was also stored in a 50 ml sampling tube with silica beads. We further identified sample 1960–3680 held in the collections of the Museúm National d’Histoire Naturelle in Paris, which is a bone from a paw of a male lion cub. Accession records show that it was collected in 1959 from the region of Franceville, Gabon in the southeast of the country (Fig. 1).

### Extraction and library amplification: skin and hair

DNA extraction from skin and hair (Table 1) was performed in a dedicated pre-PCR DNA laboratory at the Centre for GeoGenetics at the Natural History Museum of Denmark (Copenhagen). Samples were pre-digested for ca. 12 h, and then re-digested for ca. 12 h, with both steps occurring in a proteinase K containing buffer (Gilbert et al. 2007). The digest was centrifuged at 6000*g* for 1 min, after which 500 μl of supernatant was mixed 1:8 with a binding buffer as detailed in Allentoft et al. (2015) then centrifuged through Monarch DNA Cleanup Columns (5 μg) (New England Biolabs Inc. Beverly, MA, USA). DNA bound to the columns was washed with 800 μl buffer PE (Qiagen, Hilden, Germany), then eluted using two washes in 12 μl buffer EB (Qiagen)—each with an incubation of 5 min at 37 °C. The DNA extracts were subsequently built into genomic libraries using the BEST-single tube protocol (Carøe et al. 2017), optimised for highly degraded DNA following the modifications from Mak et al. (2017). Briefly, DNA extract (16.3 µl) was end-repaired, adapter-ligated and had adapter fill-in reaction in the same tube. After fill-in the reactions were purified over Monarch DNA Cleanup Columns (5 μg), using 750 µl buffer PB (Qiagen), washed with 800 μl buffer PE (Qiagen) and then eluted using two washes in 19 μl buffer EB (Qiagen)—each with an incubation of 5 min at 37 °C. We then amplified the DNA in a 50 µl reaction, using 5 µl of library under the following reaction conditions. Final concentrations were, 0.2 mM dNTPs (Invitrogen, Carlsbad, CA, USA), 2.5U PfuTurbo Cx hotstart DNA polymerase (Agilent Technologies, Palo Alto, CA, USA), 1X PfuTurbo Cx reaction buffer (Agilent Technologies), 0.4 mg/ml BSA (New England Biolabs Inc), 0.2 µM of each forward (Illumina InPE 1.0 forward) and custom made reverse primers, and 33 µl AccuGene molecular biology water (Lonza, Basel, Switzerland). PCR cycling conditions were: initial denaturation at 95 °C for 2 min followed by 26 cycles of 95 °C for 30 s, 60 °C for 30 s and 68 °C for 30 s, and a final elongation step 68 °C for 7 min.

**Table 1:** Samples analysed in this study

#	Origin	Country	Element	Collection Date	Identity	Mtgenome coverage
1	Plateaux Batéké NP	Gabon	Hair	2016	Lion	17×
2	Plateaux Batéké NP	Gabon	Hair	2016	Lion	44×
3a	Plateaux Batéké NP	Gabon	Hair	2016	Unknown	5×
3b	Plateaux Batéké NP	Gabon	Hair	2016	Unknown	No seq.
4	Plateaux Batéké NP	Gabon	Hair	2016	Bovid	0.5×
5	Odzala-Kokoua NP	Congo	Skin	1995	Lion	52×
6	Franceville (1960–3680)	Gabon	Bone	1959	Lion	0.98×

### Extraction and library amplification: bone

Bone extraction (Table 1) was performed in the same laboratory at the Centre for GeoGenetics. Extraction was performed as described by Orlando et al. (2013) in parallel with negative extraction controls. The DNA extract and negative control were then built into genomic libraries using the NEB E6070 kit (New England Biolabs), following a protocol slightly modified from that by Vilstrup et al. (2013). Briefly, extract (30 µl) was end-repaired and then passed through a MinElute column (Qiagen). The collected flow-through was then adapter-ligated and passed through a QiaQuick column (Qiagen). Adapter fill-in reaction was then performed on the flowthrough, before final incubation at 37 °C (30 min) followed by inactivation overnight at − 20 °C.

We then amplified the DNA in a 50 µl reaction, using 25 µl of library for 12 cycles under the following reaction conditions. Final concentrations were 1.25U AccuPrime™ Pfx DNA Polymerase (Invitrogen), 1 × AccuPrime™ Pfx reaction mix (Invitrogen), 0.4 mg/ml BSA, 120 nM primer in PE, and 120 nM of a multiplexing indexing primer containing a unique 6-nucleotide index code (Illumina, San Diego, CA, USA).

### Post-PCR

Post-PCR, libraries were purified with QiaQuick columns (Qiagen) and eluted with 30 µl EB after an incubation for 10 min at 37 °C. Post amplification libraries were analysed on an Agilent 2200 TapeStation HS chip (Agilent Technologies) for fragment size estimation and molar concentration was assayed. Quantified libraries were communally pooled in equimolar ratios and sequenced as single-end reads (100 bp) on an Illumina HiSeq2000 platform at the Danish National High-Throughput Sequencing Centre.

### Sequence processing

Post-sequencing read processing was performed using the PALEOMIX pipeline (Schubert et al. 2014). Adaptor removal and trimming of low quality bases was done in AdapterRemoval v2.0.0 (Schubert et al. 2016), removing reads shorter than 30 bp. All retained reads were mapped against the leopard reference genome (Wei et al. 2011), as well as 15 other mammalian mitogenomes (see Supplementary Table) with BWA-MEM v0.7.5a (Li 2013). Finally, we used Picard v1.140 (http://broadinstitute.github.io/picard/) to identify and filter PCR duplicates by the 5′-end mapping coordinate.

### Phylogenetic analysis

For each sample, we obtained the consensus sequence against the *Panthera leo* reference with ANGSD (“-minMapQ 20 -minQ 20 -doCounts 1 -setMinDepth 5 -doFasta 2”) and, together with the *Panthera leo* reference, extracted the *cyt b* (cytochrome *b*) region. These sequences, together with the Franceville Lion, were added to a pre-existing alignment of *cyt*-*b* from (Barnett et al. 2014) using MAFFT-GINSI v7.305b option “-add” (Katoh and Standley 2013). From the resulting alignment, we built a NJ phylogenetic tree with FastME v2.1.5 (Lefort et al. 2015) assuming the F84 DNA model, doing SPR tree topology improvement, and 1000 bootstrap trees.

## Results

**Fig. 3:**
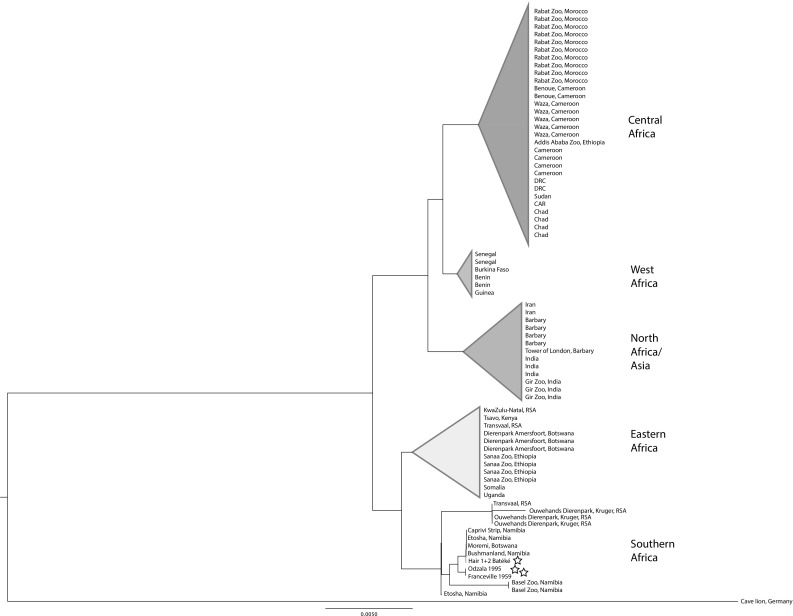
Phylogenetic tree of cytochrome *b* for lions showing in detail the position of the lions from Plateaux Batéké, Odzala-Kokoua, and Franceville identified by stars. Other populations coloured in accordance with Fig. 1. Central lineage = Red; Western lineage = Orange; Northern lineage = blue; Eastern lineage = yellow. (Color figure online)

One hair library (3a) failed to PCR amplify, indicating the source DNA was too poor for analysis. However libraries built on the remaining 4 hairs, and skin and bone samples were successfully sequenced (Table 1). Hairs 1 and 2 yielded complete mitochondrial genomes that confirm their origin to lion, and given their sequence identity and the paucity of lions in the region, it is likely they derive from the same individual. In contrast, no conclusive results could be obtained from hairs 3 and 4, in which the DNA was either too degraded to produce lion sequence, or came from a different mammal species. A complete mitogenome was also generated from the Odzala skin sample, and the Franceville specimen for comparison. Given a large comparative data set of ancient and modern lions from throughout their historic range exists for the mitochondrial *cyt b* gene (Barnett et al. 2014), we extracted this region from the newly generated mitogenomes. Analysis of this dataset clearly indicates that the Batéké samples group with lions from Namibia and Botswana to the south (Fig. 3), with a sequence equivalent to haplotype i in Dubach et al. (2013). The lions from Odzala and Franceville are identical, differing by only one mutation (99.9% identity) from the Batéké hair specimens and nesting within the clade of lions from southern Africa. Intriguingly, none of the lions we tested that originated from Gabon or Republic of the Congo show any affinity to lions from the geographically more proximate countries of Cameroon, Central African Republic, or Democratic Republic of the Congo, all of which exhibit a typically central African haplotype (Barnett et al. 2014).

## Discussion

Our genetic data suggests it is likely that the male lion, which is still currently present in PBNP (last camera track record on September 15th, 2017), is closely related to individuals killed in this landscape in previous decades. The fact that a highly similar mitochondrial haplotype of lion was present in the country in 1959 suggests continual low-level dispersal from a nearby source population. The slight difference between the modern Gabon lion and the modern Odzala lion could potentially be attributed to fairly high genetic diversity within the ancestral lion population of the Batéké landscape—a contiguous forest-savannah mosaic stretching across 300,000 km^2^ in southeast Gabon, southern Republic of Congo and the southwestern Democratic Republic of Congo (DRC). An alternative possibility is that the PBNP lion is the result of recent long-distance dispersal from a southern African population, somewhere in the region of Namibia and Botswana, where close maternal relatives are still found today. However, on the balance of probability we tentatively conclude that the Gabon male is likely a survivor of the ancestral Batéké lion population. Field evidence from Gabon, neighbouring Congo and southwest DRC suggests that he may be the last survivor.

An intriguing finding is the association of the haplotypes present in Gabon and Odzala with mostly southern African lions from the regions of Namibia and Botswana instead of geographically closer Central African Republic (CAR) or Cameroon (a sequence available on GenBank and claimed as from Angola was not used in the analyses because doubts have been raised as to its provenance). As dense forests appear to act as natural barrier for lions (Yamaguchi et al. 2004; Barnett et al. 2006b) it is more likely that lions reached Gabon through the mostly contiguous open habitats between Gabon and southern Africa rather than penetrating the dense forests between Gabon and Cameroon/CAR. This pattern does not appear to hold for the leopard, a habitat generalist, where haplotypes from Gabon are closest to those from the neighbouring Cameroon in spite of dense forests between them (Anco et al. 2017). This finding is in line with regional affinities found in other mammal species, which prefer more open habitats and are currently found in the Gabon/Congo savannas. The reedbuck (*Redunca* spp.) species present in southwest Gabon, for example, is the southern reedbuck (*Redunca arundinum*) and not the Bohor reedbuck (*Redunca redunca*) which occupies the Sahelo-Sudanian savannahs of Cameroon, Central African Republic (CAR) and northern DRC. It is difficult to base conclusions on the analysis of a single haploid marker, but lions, where females are the philopatric sex, may show stronger phylogeographic structure within their mitochondrial diversity. Small-scale comparisons of mitochondrial and nuclear datasets have shown that both produce similar phylogenies (Bertola et al. 2015). Nonetheless, similar problems related to population extirpation and reintroduction in another apex felid, the tiger (*Panthera tigris*), have centred almost entirely around discussion of mitochondrial haplotype datasets of equivalent size. The recently extinct Caspian tiger (*Panthera tigris virgata*) has been identified by mitochondrial DNA as belonging to a maternal lineage close to the extant Siberian tiger (*P. t. altaica*) and it has been argued that this population should be used for potential reintroduction projects (Driscoll et al. 2009, 2012).

We consider it justified to base our choice of a suitable source population for eventual release into Batéké on the results of the present analysis. Based on these, new founder individuals should be sourced from Botswana or Namibia, given the extinction of the Congo population. Potential animals for translocation should be haplotyped before moving to ensure a match to Gabonese specimens. We would recommend the wider Okavango ecosystem as the specific origin, due to certain ecological similarities such as the relatively wet conditions during parts of the year.

## Conclusion

Use of minute, degraded field samples now allows access to large volumes of genetic data. For elusive, rare, or difficult to access populations the techniques first pioneered in ancient DNA studies will continue to have relevance for including them within wider phylogeographic studies that are crucial for proper understanding of the conservation and challenges they face.

### Availability of supporting data

Sequence data produced for this study have been uploaded to GenBank, with accession numbers MG772937, MG792275, MG792276, MG792277. Raw data have been uploaded to the Sequence Read Archive at NCBI under BioProject number: PRJNA431047.

## Electronic supplementary material

Below is the link to the electronic supplementary material.

Supplementary material 1 (XLSX 47 KB)
